# A clinically relevant pharmacokinetic interaction between cyclosporine and imatinib

**DOI:** 10.1007/s00228-016-2038-9

**Published:** 2016-03-11

**Authors:** Ferdows Atiq, Annoek E C Broers, Louise M Andrews, Jeanette K Doorduijn, Birgit C P Koch, Teun Van Gelder, Jorie Versmissen

**Affiliations:** Department of Hospital Pharmacy, Erasmus Medical Center, Rotterdam, The Netherlands; Department of Hematology, Erasmus Medical Center, Rotterdam, The Netherlands; Department of Internal Medicine, Erasmus Medical Center, PO Box 2040, 3000 CA Rotterdam, The Netherlands

**Keywords:** Cyclosporine, Imatinib, Hematopoietic stem cell transplantation, Graft-versus-host disease

## Abstract

**Purpose:**

Cyclosporine A (CsA) and imatinib are both CYP3A4 and P-glycoprotein substrates. Concomitant use after hematopoietic stem cell transplantation (HSCT) for chronic myeloid leukemia (CML) or Philadelphia chromosome-positive (Ph+) acute lymphatic leukemia (ALL) may therefore result in a pharmacokinetic interaction. Although case reports and a recent small study in children indeed suggested there is a relevant pharmacokinetic interaction, a larger study in adults is lacking. In this study, we assessed the presence and extent of this interaction in patients with CML or Ph+ ALL undergoing HSCT.

**Methods:**

From a large database containing data of all patients receiving HSCT in our center between 2005 and 2015, we selected 16 patients using this drug combination. The average dose-corrected CsA concentration was calculated before and after initiation of imatinib.

**Results:**

The average dose-corrected CsA concentration increased during imatinib use in all patients, on average by 94 % (*p* < 0.001). Based on measured drug concentrations, the CsA dosage needed to be reduced, on average, by 27 % after initiation of imatinib (*p* = 0.004).

**Conclusions:**

Imatinib significantly increases CsA concentrations in HSCT patients, putting these patients at increased risk of CsA toxicity. We recommend intensive monitoring of CsA concentrations after initiation of imatinib; a pre-emptive CsA dose reduction of 25 % might be considered.

## Introduction

Cyclosporine A (CsA) is an immunosuppressive agent with a narrow therapeutic index, which acts primarily on T-helper cells [1]. It is one of the cornerstone drugs in the prevention and treatment of graft-versus-host disease (GvHD) after hematopoietic stem cell transplantation (HSCT) [1]. Imatinib is a tyrosine kinase inhibitor directed against the BCR-ABL fusion enzyme, which is present in chronic myeloid leukemia (CML) and Philadelphia chromosome-positive acute lymphoid leukemia (Ph+ ALL) [2–4]. The drug combination CsA and imatinib is a possible treatment combination after HSCT for CML and Ph+ ALL. In these Ph+ leukemias, imatinib is initiated or re-initiated after bone marrow repopulation to further prevent recurrence of the disease [5]. Both CsA and imatinib are substrates as well as inhibitors of CYP3A4 and P-glycoprotein (P-gp) [1, 3]. Therefore, inhibition of CYP3A4 and/or P-gp by imatinib may increase CsA exposure and vice versa [6, 7]. In view of the narrow therapeutic index of CsA, and as many patients following HSCT already suffer from some degree of renal insufficiency, it is important to avoid supra-therapeutic CsA exposure.

Some case reports showed CsA toxicity during imatinib use, potentially as a consequence of this interaction [8, 9]. In a recent case series, a higher CsA exposure was found after 3 to 7 days of imatinib therapy in six children undergoing HSCT [10]. However, data in adults and recommendations for adjusting the dosage of CsA are lacking. Based on our own clinical observations and these earlier smaller studies, we hypothesized that this interaction between CsA and imatinib leads to increased concentrations of CsA requiring dose adjustment. Therefore, we assessed CsA-imatinib interaction in patients after HSCT by measuring dose-corrected CsA concentrations before and after initiation of imatinib.

## Methods

We performed a retrospective cohort study in CML and Ph+ ALL patients who underwent HSCT. Patients were selected from a database of 44 patients, containing all patients undergoing HSCT between 2005 and 2015 in the Erasmus Medical Center because of CML or Ph+ ALL. We used the electronic patient records to scan the patients for eligibility and to collect data.

The inclusion criteria were simultaneous use of oral CsA and imatinib and at least one CsA concentration in the month before and at least one CsA concentration after imatinib initiation. The only exclusion criterion was use of azole antifungals (i.e. fluconazole, voriconazole and itraconazole) only before or only after initiation of imatinib, because azole antifungals are CYP3A4 and P-gp inhibitors and therefore increase CsA concentrations during concomitant use [11]. Patients were included if the azole antifungal was used during the whole study period.

Taken into account the CsA intrapatient variability, we aimed to collect for each patient five CsA trough concentrations before and five trough concentrations after initiation of imatinib treatment. The used target level for this indication was 250–350 ng/mL. The CsA assay was performed using a validated UPLC-MS/MS method, routinely used in our clinic.

The CsA concentration measurement was excluded from the analysis if the CsA dosage used at that time point could not be obtained from the patient records or if the treating physician had documented uncertainty regarding the time of drug intake, i.e. whether the measured concentration was indeed a trough level.

### Analysis

We calculated the dose-corrected CsA concentration for each concentration measurement by dividing the CsA concentration (ng/mL) by the CsA dosage (daily dose) used at that moment. The average dose-corrected CsA concentration was calculated for each patient before and after initiation of imatinib. We compared these averages by using a paired sample *t* test. We calculated the effect of different imatinib dosages on dose-corrected CsA concentrations by comparing the difference between average dose-corrected CsA concentrations before and during imatinib for each imatinib dosage using a linear regression model. Significance was defined as *p* < 0.05.

No formal ethical approval was obtained since the research was retrospective using anonymized data. All patients signed informed consent when undergoing HSCT including information about the use of anonymized medical data by scientists.

## Results

We included 16 patients out of 44 patients: in five patients, data (especially exact CsA doses; one patient lost to follow-up) were missing, 18 patients did not receive imatinib after HSCT (three died because of complications of HSCT, 13 switched to another tyrosine kinase inhibitor after HSCT due to imatinib refractory disease), three patients did not receive CsA at the moment imatinib was re-initiated and two patients received an azole antifungal only after initiation of imatinib. Table 1 shows the patient characteristics. On average, there were 4.2 CsA concentration measurements both before and after initiation of imatinib treatment. The first CsA measurement taken into account was on average 20.4 days (standard deviation (SD) 12.6) before initiation of imatinib and the last measurement 21.8 days (SD 12.4) after (Table 2). In two patients, the imatinib dosage was changed during the study period. All others used a stable daily dose of 400 or 600 mg.

**Table 1:** General characteristics of included patients

Patient	Sex	Age	Weight (kg)	Baseline creatinine (μmol/L)^a^	Diagnosis	Donor	Conditioning	Imatinib (mg)
1	M	17	66	98	ALL	SIB	Myeloablative	400
2	M	56	68.1	126	ALL	MUD	RIC	400
3	F	65	62	53	ALL	MUD	RIC	400^b^
4	M	17	65.8	70	ALL	MUD	Myeloablative	600
5	F	47	64.3	71	ALL	SIB	RIC	600
6	F	17	63.4	132	CML	MUD	Myeloablative	600
7	M	21	65.5	72	ALL	UCB	RIC	600
8	F	41	56	93	ALL	UCB	RIC	600^c^
9	F	58	80.4	84	CML	MUD	RIC	400
10	F	51	85.8	78	ALL	SIB	RIC	600
11	M	35	78.8	140	CML	UCB	RIC	400
12	F	57	64.1	83	CML	UCB	RIC	600
13	M	41	75	91	ALL	SIB	RIC	600
14	F	59	46.9	66	ALL	MUD	RIC	600
15	M	62	74.7	130	ALL	MUD	RIC	600
16	F	51	76.9	91	CML	UCB	RIC	400

*M* male, *F* female, *CsA* cyclosporine A, *ALL* acute lymphatic leukemia, *CML* chronic myeloid leukemia, *SIB* sibling, *MUD* matched unrelated donor, *UCB* umbilical cord blood, *RIC* reduced intensity conditioning

^a^Measured on the same day or less than 1 week before first included CsA measurement

^b^Dose switch to 600 mg from 3rd CsA concentration

^c^Dose switch to 400 mg from 4th CsA concentration

**Table 2:** Dosages and concentrations of CsA before and after initiation of imatinib

Patient	Number of CsA concentrations		Number of days^a^		Mean daily CsA dosage (mg) (range)		Mean CsA concentration (ng/mL) (range)	
	Before	During	Before	After	Before	After	Before	After
1	5	5	42	42	150 (150)	95 (75–125)	285 (221–355)	339 (272–513)
2	2	2	2	3	225 (200–250)	50 (25–75)	358 (308–408)	228 (161–294)
3	5	5	9	14	150 (150)	80 (25–150)	263 (214–299)	411 (273–586)
4	2	5	7	38	138 (125–150)	115 (100–150)	239 (192–285)	289 (183–438)
5	5	5	23	21	200 (200)	120 (100–200)	283 (212–321)	409 (242–847)
6	5	5	22	9	225 (200–250)	160 (125–175)	259 (209–333)	315 (291–376)
7	3	4	8	14	175 (150–200)	175 (150–200)	341 (205–572)	453 (311–576)
8	5	5	24	25	125 (100–175)	130 (112.5–150)	212 (107–361)	352 (118–696)
9	5	5	28	16	190 (175–212.5)	148 (125–212.5)	283 (177–438)	271 (194–338)
10	3	5	14	18	300 (250–400)	180 (150–250)	342 (250–469)	345 (237–437)
11	4	1	12	NA	156 (100–225)	225 (225)	205 (159–280)	351 (351)
12	5	2	15	4	290 (225–325)	300 (250–350)	336 (142–561)	608 (537–678)
13	5	5	19	21	165 (150–175)	105 (75–175)	316 (232–429)	421 (332–615)
14	3	5	24	31	150 (150)	95 (50–175)	258 (236–284)	399 (207–542)
15	5	4	28	36	155 (125–175)	81 (50–100)	304 (195–495)	268 (161–393)
16	5	4	49	35	105 (75–125)	69 (25–125)	176 (134–234)	158 (77–315)

^a^Between first CsA measurement and initiation of imatinib, respectively between initiation of imatinib and last CsA measurement

The first CsA concentration after initiation of imatinib led to a rise in CsA trough concentration above the target of 350 ng/mL in 10 out of 16 patients. After the first CsA concentration, CsA was discontinued in one patient and the dose was reduced in 12 patients. In the remaining three patients, the CsA dose was reduced after the second CsA measurement after initiation of imatinib. In all patients, dose reduction of CsA was necessary after initiation of imatinib.

**Fig. 1:**
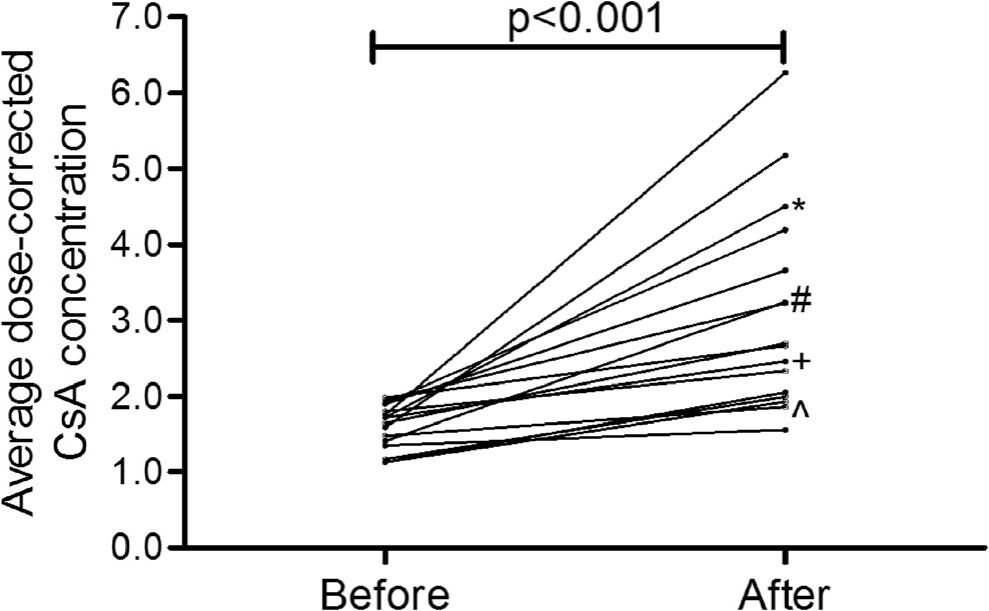
Average dose-corrected CsA concentration before and after initiation of imatinib. The average of all ratios of CsA concentration (ng/mL) divided by the used daily dose of CsA on that day (mg) before and after initiation of imatinib. Patients using *fluconazole 50 mg once daily; ^#^fluconazole 400 mg once daily; ^voriconazole 200 mg twice daily, all the same dose before and after initiation of imatinib; ^+^fluconazole 400 mg once daily before and 200 mg once daily after initiation of imatinib

The average dose-corrected CsA concentration significantly increased during imatinib by 94 % (*p* < 0.001, range 16–257 %) as shown for each patient in Fig. 1. Following dose adjustments, the average CsA dosage was 27 % lower during imatinib (*p* = 0.004, range 78 % lower–44 % higher). Overall, despite dose adjustments, the average CsA concentrations were 26 % higher during imatinib (*p* = 0.009, range 36 % lower–81 % higher). The supra-therapeutic CsA concentrations coincided with an increase of creatinine concentration (range 13–64 μmol/L increase) at the next measurement after initiation of imatinib in 13 out of 16 patients.

The mean variability in dose-corrected CsA concentrations was lower before than after initiation of imatinib (ranges shown in Table 2; *p* for difference in variability of dose-corrected CsA concentrations = 0.085).

There was no significant difference in dose-corrected CsA concentrations and the necessity of CsA dose adjustment between patients using 400 or 600 mg imatinib.

Cyclosporine pharmacokinetics might be influenced by other CYP3A4 substrates. The only CYP3A4 inhibitors used in this population were antifungal agents know as ‘–azoles’. Patients using voriconazole or fluconazole showed a rise in dose-corrected CsA concentrations comparable to patients not using these agents (as indicated with a symbol in Fig. 1). However, the rise in the only patient using voriconazole (the stronger CYP3A4 inhibitor) was less pronounced. One patient used different fluconazole dosages before and after initiation of imatinib; however, his CsA dose-corrected concentration was comparable to those of other patients on fluconazole or voriconazole. None of the patients used another CYP3A4 substrate or inhibitor, such as diltiazem, verapamil or St John’s wort.

## Discussion

In this retrospective cohort study, it is shown that imatinib significantly increases CsA exposure in CML and Ph+ ALL patients after HSCT, leading to an average dose reduction of 27 % to reach similar CsA concentrations. Therefore, pre-emptive CsA dose reduction at the time of initiation of imatinib treatment and subsequent intensive CsA concentration monitoring is recommended in order to avoid additional CsA-related toxicity, such as nephrotoxicity which was also seen in this population.

Our results confirm earlier preliminary findings in case reports and one case series [8–10]. Two studies found no increase in CsA exposure during imatinib, but these studies both had important limitations: in the first study, the participants received a subtherapeutic imatinib dosage of 200 mg, while in the second study CsA dosages were not taken into account [5, 12].

In a recent case series in six children, the pre-emptive CsA dose reduction following imatinib co-treatment was suggested to be 40 % [10]. In our study, the average dose reduction was 27 %. There was a substantial interpatient variability in dose-corrected CsA concentrations after initiation of imatinib. CsA is well known to have a large interpatient as well as intrapatient variability caused by different factors such as differences in nutrition and genetic variability [1, 13]. We aimed to minimize the effect of intrapatient variability by aiming to collect five CsA concentrations before and five CsA concentrations after initiation of imatinib. The intrapatient variability in dose-corrected CsA concentrations was larger after initiation of imatinib than before initiation (*p* = 0.085). This emphasizes the importance of intensive CsA concentration monitoring after initiation of imatinib. These findings did not influence our conclusion, because it does not explain the significant increase in average dose-corrected CsA concentrations.

The increase in CsA concentrations coincided with an increase of creatinine concentration at the next measurement after initiation of imatinib. This study was underpowered for this endpoint. However, the observed rise in creatinine concentrations does emphasize the importance of avoiding supra-therapeutic CsA exposure. In two patients, CsA treatment was discontinued or interrupted after initiation of imatinib due to progressive renal insufficiency.

Patients using CYP3A4 inhibitors such as voriconazole or fluconazole showed a rise in dose-corrected CsA concentrations comparable to patients not using these agents (as indicated with an asterisk in Fig. 1). However, the rise in the only patient using voriconazole (the stronger CYP3A4 inhibitor) was less pronounced.

The mechanism of the pharmacokinetic interaction between CsA and imatinib is most likely inhibition of CYP3A4 activity by imatinib. Potentially, the interaction may also be explained by increased bioavailability of CsA due to imatinib-induced inhibition of the P-glycoprotein efflux pump in enterocytes [14]. We anticipated that concomitant use of other CYP3A4 substrates might influence the result of adding imatinib to CsA [15]. Four patients used voriconazole (*n* = 1) or fluconazole (*n* = 3), both before and after initiation of CsA. Although we expected that in these patients the pharmacokinetic interaction between CsA and imatinib would be less pronounced, we did see a rise in dose-corrected CsA concentrations comparable to patients not using these agents (as indicated with a symbol in Fig. 1). However, the only patient with voriconazole co-treatment (the stronger CYP3A4 inhibitor) had a rise in average dose-corrected CsA concentration from only 1.48 before to 1.86 after initiation of imatinib.

The strength of our study is that it is the first study in a relatively large group of HSCT patients aged >16 years old that shows increased CsA exposure after initiation of imatinib.

A potential limitation is the retrospective observational study design. However, the medication data and laboratory measurements were well documented in the patient files. In our view, the sample size is sufficiently large to draw conclusions and recommend dose reductions when imatinib is added to CsA treatment, especially since the effect was consistently observed in all patients. Furthermore, the mechanism for the pharmacokinetic interaction is plausible.

Nowadays, tacrolimus is also used as immunosuppressive drug instead of CsA [16]. Since tacrolimus is also a substrate of CYP3A4 and P-gp, it will be interesting to study the effect of initiation of imatinib for tacrolimus concentrations as well. Since in our center CsA is the first choice for GvHD prevention or treatment, we could not investigate this further.

In conclusion, imatinib increases CsA exposure in CML and Ph+ ALL patients after HSCT leading to increased CsA concentrations requiring an average dose reduction of 27 %. Therefore, we recommend a pre-emptive CsA dose reduction of at least 25 % when initiating imatinib, to prevent CsA toxicity. Moreover, CsA concentrations should be closely monitored, to avoid underexposure or overexposure.

A good option would be perform therapeutic drug monitoring on the day of initiation of imatinib to enable dose adjustment by Bayesian modeling [17].

### Authorship contributions

F.A. and L.A. contributed to the design of the work, collected data and wrote the manuscript. A.B., J.D. and B.K. enabled data collection. F.A. and J.V. performed the analyses. J.V., T.V., A.B., J.D. and B.K. contributed to the design of the work and wrote the manuscript. All authors reviewed and edited the paper.
